# Decrease of alpha-crystallin A by miR-325-3p in retinal cells under blue light exposure

**DOI:** 10.1016/j.mocell.2024.100091

**Published:** 2024-07-10

**Authors:** Subeen Oh, Chongtae Kim, Young-Hoon Park

**Affiliations:** 1 Catholic Institute for Visual Science, College of Medicine, The Catholic University of Korea, Seoul 06591, South Korea; 2 Department of Ophthalmology, College of Medicine, The Catholic University of Korea, Seoul 06591, South Korea

**Keywords:** Alpha-crystallin A, High-energy visible light, MicroRNAs, Neuro-retinal cell, Retina

## Abstract

Exposure to blue light can lead to retinal degeneration, causing adverse effects on eye health. Although the loss of retinal cells due to blue light exposure has been observed, the precise molecular mechanisms underlying this process remain poorly understood. In this study, we investigate the role of alpha-crystallin A (CRYAA) in neuro-retinal degeneration and their regulation by blue light. We observed significant apoptotic cell death in both the retina of rats and the cultured neuro-retinal cells. The expressions of *Cryaa* mRNA and protein were significantly downregulated in the retina exposed to blue light. We identified that miR-325-3p reduces *Cryaa* mRNA and protein by binding to its 3′-untranslated region. Upregulation of miR-325-3p destabilized *Cryaa* mRNA and suppresses CRYAA, whereas downregulation of miR-325-3p increased both expressions. Blue light-induced neuro-retinal cell death was alleviated by CRYAA overexpression. These results highlight the critical role of *Cryaa* mRNA and miR-325-3p molecular axis in blue light-induced retinal degeneration. Consequently, targeting CRYAA and miR-325-3p presents a potential strategy for protecting against blue light-induced retinal degeneration.

## INTRODUCTION

With the advancement of lighting technologies, our lives have become increasingly exposed to light-emitting diodes (LEDs) through various sources, such as computer monitors, smartphones, and lamps (Ouyang et al., 2020, Sliney, 2016). White light is commonly produced by combining blue LEDs with yellow phosphor coatings due to its cost-effectiveness and popularity (Krigel et al., 2016). LED light contains a significant amount of blue radiation, which poses a potential risk to the retina. Blue light, characterized by its short wavelength in the range of 450 to 495 nm, is a part of the high-energy visible light spectrum compared to other colors (Kuse et al., 2014). It is well-established that short-wavelength blue light can induce cell death in retinal neurons by generating oxidative stress and causing mitochondrial dysfunction (Yang et al., 2021, Yoshida et al., 2015). Retinal degeneration is a progressive neurodegenerative disorder characterized by the apoptotic loss of retinal cells, caused by environmental or genetic factors (Athanasiou et al., 2013, Hao et al., 2002). Specifically, exposure to intense visible light of approximately 500 nm wavelength (blue light) can lead to harmful effects on retinal tissues by causing photochemical damage (Athanasiou et al., 2013, Jaadane et al., 2015, Paskowitz et al., 2006). While the injection of vascular endothelial growth factor inhibitors or photodynamic therapy are currently the most effective treatments for delaying the progression of retinal degeneration (AIELLO, 1995), there is still no effective cure available.

Crystallins are predominantly expressed structural proteins in the lens, and they play roles in maintaining the transparent and refractive properties of the lens (Bloemendal and de Jong, 1991, Fort and Lampi, 2011, Wistow and Piatigorsky, 1988). The expression of crystallins has also been demonstrated not only in the retina, cornea, optic nerve, astrocytes, and Müller cells but also in nonocular tissues (Piri et al., 2013, Slingsby and Wistow, 2014, Srinivasan et al., 1992). Crystallins, which consist of 3 major families (α, β, and γ), are essential proteins in the eyes (Andley, 2007). The α-crystallin shares homology with small heat shock proteins and has chaperone-like properties, including resistance to cellular stress (Piatigorsky, 1998). α- and β-crystallins have been implicated in retinal neuroregeneration in conditions such as stroke, endophthalmitis, and uveitis, whereas the role of γ-crystallins in the retina is less understood (Arac et al., 2011, Rao et al., 2012, Rao et al., 2008, Ruebsam et al., 2018, Whiston et al., 2008). A recent study demonstrated that α- and β-crystallins are downregulated in photoreceptor cells injured by blue light, suggesting that crystallins may play a neuroprotective role in the retina (Heinig et al., 2020). However, the detailed molecular mechanisms regulating crystallin expression remain unclear.

MicroRNAs (miRNAs) are a class of small noncoding RNAs composed of approximately 22 nucleotides (Bartel, 2004). MiRNAs function to downregulate protein expression by controlling the stability or translation of target mRNAs by binding to complementary sequences on the mRNA molecules (Bartel, 2004, Loscher et al., 2008). MiRNAs play critical roles in the regulation of various cellular processes, including cell proliferation, differentiation, apoptosis, and division, and have implications for eye diseases (Croce and Calin, 2005, He and Hannon, 2004, Kim et al., 2018). The expression of specific miRNAs has been associated with retinal diseases such as retinal degeneration and diabetic retinopathy (Mastropasqua et al., 2014). Several studies have demonstrated that the regulation of miR-124, -223, -144, and -155 contributes to the protection of retinal function by reducing inflammation and cell death (Aggio-Bruce et al., 2021, Chu-Tan et al., 2018, Fernando et al., 2020, Jadeja et al., 2020). For instance, blue light exposure reduces miR-22-3p, leading to an increase in NLRP3 expression in retinal pigment epithelial (RPE) cells (Hu et al., 2019). Furthermore, in RPE cells exposed to blue light-induced oxidative stress, miR-27a promotes cell death by targeting *FOXO1* mRNA (Ren et al., 2021). However, no study has investigated the miRNAs responsible for the development of neuro-retinal cell degeneration.

In this study, we investigated a novel mechanism involved in neuro-retinal degeneration induced by blue light. We observed that alpha-crystallin A (CRYAA) was downregulated in the retinal tissues of rats and in neuro-retinal cells exposed to blue light, and this downregulation was mediated by miR-325-3p. Our results suggest that the molecular interaction between *Cryaa* mRNA and miR-325-3p plays a crucial role in blue light-induced neuro-retinal degeneration. Therefore, targeting CRYAA and miR-325-3p may offer a promising approach to protect against retinal degeneration caused by blue light.

## MATERIALS AND METHODS

### Animals

Six-week-old male albino Wistar rat was used in this study. The rats were purchased from Orient Bio (Seongnam, Korea). Rats were maintained on a 12-hour/12-hour light-dark cycle at 20 to 26 °C, 50 ± 10% relative humidity. Food (gamma lay sterilized diet TD 2018S; Harlan Laboratories) and water (autoclaved R/O) were available ad libitum. The rats were treated humanely in accordance with the Association for Research in Vision and Ophthalmology Statement for the use of animals in Ophthalmic and Vision Research, as well as the Laboratory Animals Welfare Act, the Guide for the Care and Use of Laboratory Animals, and the Guidelines and Policies for Rodent experiments provided by the Institutional Animal Care and Use Committee at the School of Medicine, The Catholic University of Korea (Approval number: CUMS-2021-0230-03). All experimental procedures followed these guidelines and regulations. The study was carried out in compliance with the Animal Research: Reporting of In Vivo guideline. Institutional Animal Care and Use Committee and the Department of Laboratory Animal at the Catholic University of Korea, Songeui Campus, accredited the Korea Excellence Animal Laboratory Facility from Korea Food and Drug Administration in 2017 and reaccredited in 2021, and also acquired The American Association for Assessment and Accreditation of Laboratory Animal Care International full accreditation in 2018 and reaccredited in 2022.

### Cell Culture, Treatment and Transfection of siRNA, Precursors and Inhibitors of miRNA

R-28 rat retinal neuronal cells were grown in Dulbecco’s Modified Eagle’s Medium (WelGENE) supplemented with 10% fatal bovine serum (HyClone), 1% penicillin (WelGENE) in a humidified atmosphere of 5% CO_2_ at 37 °C. R-28 cells were cultured on 10-cm cell culture dishes (SPL Life Sciences) and the medium was replaced every 2 to 3 days. All transfections including control small interfering RNA (siCtrl), precursor and inhibitor of miR-325-3p (Genolution), and plasmid, pCMV-Ctrl, myc tagged Cryaa (pCMV-Cryaa) (OriGene), and enhanced green fluorescent protein (EGFP) reporter plasmids were done using with Lipofectamine 2000 (Invitrogen). EGFP reporter was cloned by inserting 3′ untranslated region (UTR) of *Cryaa* mRNA into the pEGFP-C1 (BD Bioscience), respectively.

### Light Source and Exposure

For blue light exposure, specific devices were built by CMLAB Co. The day before light exposure, rats were dark-adapted overnight (16 hours). The pupils were dilated with one drop of 0.5% tropicamide and 0.5% phenylephrine hydrochloride (Mydrin-P; Santen Pharmaceutical) 30 minutes before the exposure to blue light. The nonanesthetized rats were exposed to 2,000 lux of the blue light (460 nm) for 2 hours. Blue light irradiation with an output power of 20 mW/cm^2^ was applied delivering 144.5 J/cm^2^ of energy. Control rats were submitted to the same preconditioning protocol, but not exposed to light. Exposure intensity was spectrophotometrically measured by the light meter (TM-201; TENMARS). After exposure, rats were placed again in a cyclic light/dark (250 lux, 12 hours/12 hours) environment for 3 days, followed by sacrificing for histological analysis and RNA extraction. Blue-light irradiation of R-28 cells was performed by modifying as described above. Cells were irradiated with 460 nm light for 10 minutes at 20 mW/cm^2^ (12 J/cm^2^). The distance between the light source and cell plates was 8 cm. Irradiation had no effect on the temperature of the cell medium.

### RNA Analysis

Total RNA was prepared from whole cells using RNAiso Plus (TaKaRa). After reverse transcription using ReverTra Ace qPCR RT Master Mix (Toyobo), abundance of transcripts was assessed by real-time quantitative PCR (RT-qPCR) analysis using SensiFAST SYBR No-ROX Mix (Bioline) and gene-specific primer sets (Table 1). RT-qPCR was performed on CFX Connect Real-Time System (Bio-Rad). Individual miRNAs were further quantified using Mir-X miRNA First Strand Synthesis Kit (Takara Bio Inc). For Mir-X cDNA analysis, forward primers were designed to be the exact sequences of miRNAs based on the miRBase database, and a universal reverse primer provided by the kit was used.

**Table 1:** Primer sequences used in this study

*Primers for EGFP-reporter*	*Sequences*		
rat *Cryaa*-3U-F	5′-AAAAAGATCTTAAGCAGGCCTCGCCTTGG-3′		
rat *Cryaa*-3U-R	5′-AAAAGGTACCGCTTGTCACCTGCTCT-3′		
rat *Cryaa*-3U-MUT	5′-GGAGCCCCUGGCAGAGUUAUUAG-3′		
			
*Primers for PCR*	*Sequences*	*Product size*	*References (primer bank number)*
rat *Cryaa*-F	5′-CCTGCTGCCCTTCCTGTCGT-3′	210 bp	DOI: 10.1016/j.bbrc.2018.06.149
rat *Cryaa*-R	5′-TCCTGGCGCTCGTTGTGCT-3′		
rno-miR-325-3p	5′-UUUAUUGAGCACCUCCUAUCAA-3′	22 bp	*N.S.*
rat *Gapdh*-F	5′-TGCCACTCAGAAGACTGTGG-3′	123 bp	DOI: 10.1523/JNEUROSCI.1436-10.2010
rat *Gapdh*-R	5′-TTCAGCTCTGGGATGACCTT-3′		

### Western Blot Analysis

Whole-cell lysates were prepared using RIPA buffer (10 mM Tris-HCl [pH 7.4], 150 mM NaCl, 1% NP-40, 1 mM EDTA, and 0.1% SDS), separated by SDS-PAGE, and transferred onto polyvinylidene difluoride membranes (Millipore). Protein concentrations were measured using Bradford Protein Assay Kit (Bio-Rad) with bovine serum albumin (BSA) as a standard. Incubation with primary antibodies to detect Cryaa (Santa Cruz Biotechnology Inc), β-actin (Abcam), and EGFP (Santa Cruz Biotechnology Inc) was followed by further incubation with the appropriate secondary antibodies conjugated with horseradish peroxidase (Santa Cruz Biotechnology Inc) and detection using WestGlow FEMTO ECL Chemiluminescent Substrate Kit (Biomax).

### Cell Viability Assay

The cell viability was measured by the reduction of 3-(4,5-dimethylthiazol-2-yl)-2,5-diphenyltetrazolium bromide (MTT, Invitrogen). The R-28 cells were seeded into 96-well plates and then incubated for 24 hours under a humidified atmosphere of 5% CO_2_ at 37 °C. Next, MTT solution (5 mg/ml) was added to each well, and the cells were incubated for 2 hours at 37 °C. Afterward, the medium was removed, and MTT was solubilized by adding 100 µl of 0.04 N HCl-isopropanol and 100 µl of distilled water to each well. After agitation of the plates for 10 minutes, the optical density of the solubilized crystals at 570 nm was measured using an automated microplate reader. The average absorbance of controls in each experiment was defined as 100%.

### Histological Analysis

Following 3 days of experimentation, the eyes of albino Wistar rats euthanized with CO_2_ were removed carefully and the anterior parts and lens were discarded (n = 8 for each group). Eyes were fixed overnight in 4% paraformaldehyde and immersed in 30% sucrose for 24 hours. The eyes were then embedded in optimal cutting temperature compound (Sakura) and stored at −80 °C until the sectioning. A cryo-microtome was used to cut 7 µm retinal sections at −20 °C and stored at −80 °C until staining. The stained sections were photographed using a virtual microscope (Olympus).

### Terminal Deoxynucleotidyl Transferase dUTP Nick End Labeling Assay

Cell death was observed using a terminal deoxynucleotidyl transferase dUTP nick end labeling (TUNEL) assay with the in situ cell death detection kit, tetramethylrhodamine red (Roche). Cells were fixed with 4% paraformaldehyde, permeabilized using 0.1% Triton X-100, and incubated with the TUNEL enzyme and labeling solution for 1 hour at 37 °C in the dark. Nuclei were stained with 4′,6-diamidino-2-phenylindole (Thermo Scientific). TUNEL-positive fluorescence was measured using a fluorescence microscope (Zeiss AXIO Observer Z1 Inverted; Carl Zeiss).

### Immunohistochemistry

Cryosections of the eyes were air-dried and washed twice with PBS. The slides were washed with 0.5% H_2_O_2_ in MeOH for 30 minutes, and twice with PBS. Sections were blocked nonspecific binding activity with 2% BSA in PBS for 1 hour. Then, slides were incubated with the primary antibodies diluted in 2% BSA overnight at 4 °C. The primary antibodies were anti-CRYAA (Santa Cruz Biotechnology). After washing 3 times for 5 minutes with PBS, the enzyme conjugated with a secondary antibody diluted in PBS on the slides was applied for 1 hour at room temperature.

### Statistical Analysis

Data are expressed as the mean ± SEM of 3 independent experiments. The statistical significance of data was determined via the Student’s *t*-test (*P < .05; **P < .01, ***P < .001).

## RESULTS

### Induction of Neuro-retinal Cell Death Under Blue Light Exposure

**Fig. 1:**
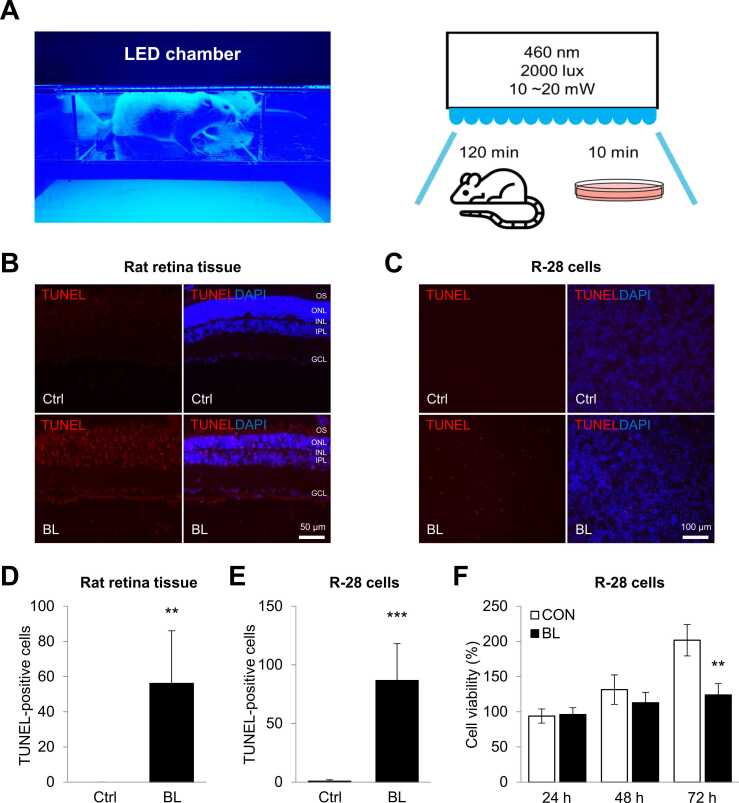
Blue light induces cell death in neuro-retinal cells. (A) Rat and R-28 cells were exposed with or without blue light (460 nm) in a chamber set at 2,000 lux for 120 minutes and 10 minutes, respectively. The sacrifice was performed 3 days after the blue light exposure. (B, C) Representative images of retina tissues and R-28 cells stained with TUNEL (Red) and DAPI (blue) after blue-light irradiation. TUNEL-positive cells are observed throughout the entire retinal layer, including the GCL. Scale bars represent 50 µm and 100 µm. (D, E) Quantitative analysis for the number of TUNEL-positive cells. (E) The viability of R-28 cells exposed to blue light or not was determined by MTT assay. **P < .01; ***P < .001. DAPI, 4′,6-diamidino-2-phenylindole*;* GCL, ganglion cell layer*.*

To understand the molecular mechanism behind blue light-induced degeneration of neuro-retinal cells, we exposed rat retinal tissues and neuro-retinal R-28 cells to blue light using an LED chamber adjusted to 460 nm and 2,000 lux (Fig. 1A). First, Wistar rats were exposed to blue light for 2 hours, whereas control rats were kept in the chamber without exposure. Cell death in blue light-exposed rat retinal tissues was analyzed by TUNEL staining. TUNEL-positive cells in the ganglion cell layer, inner nuclear layer, outer nuclear layer, and outer segments of retinal tissue were significantly induced by blue light exposure (Fig. 1B and D). Next, R-28 cells were exposed to blue light for 10 minutes, and cell death and cell viability were determined using TUNEL and MTT assays, respectively. TUNEL-positive R-28 cells were significantly increased (Fig. 1C and E), and moreover, the viability of R-28 cells was significantly reduced compared to that of control cells, 72 hours after exposure to blue light (Fig. 1F). Therefore, these results indicate that blue light negatively affects the cell death of both rat retinas and cultured neuro-retinal cells.

### Downregulation of CRYAA Expression by Blue Light in Neuro-retinal Cells

**Fig. 2:**
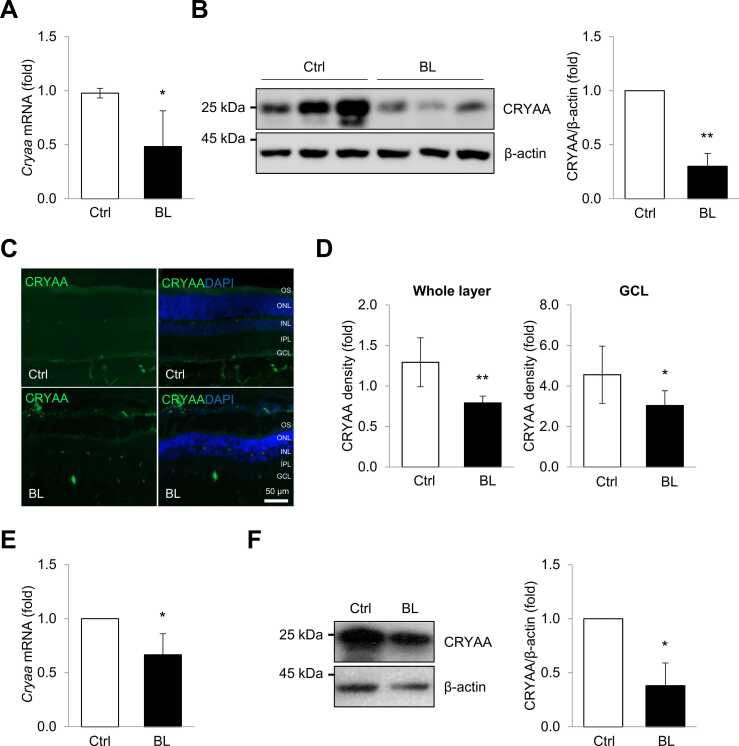
Exposure to blue light caused reducing the expression of *Cryaa* mRNA and protein. (A, B) After blue light exposure, levels of *Cryaa* mRNA and protein in the retinal tissues of rats were analyzed by RT-qPCR and western blotting. (C) Representative immunoreactivity of CRYAA in the retinal tissues of rats exposed to blue light. CRYAA (green) is expressed in the GCL and OS surrounding the nuclei of neuro-retinal cells (DAPI: nuclear staining, blue). (D) A quantitative analysis of the density of CRYAA is shown. (E, F) levels of *Cryaa* mRNA and protein in blue light exposure R-28 cells were determined by RT-qPCR and western blotting. *Gapdh* mRNA was used for normalization, and β-actin was used as a loading control. Data represent the means ± SEM from 3 independent experiments*.* *P < .05; **P < .01. DAPI, 4′,6-diamidino-2-phenylindole*;* GCL, ganglion cell layer; OS, outer segments*.*

Although the impact of blue light on the survival and function of neuro-retinal cells has been recognized, the precise molecular mechanisms and responsible factors have not yet been fully elucidated. A recent study revealed an association between several crystallins and the development of neurodegeneration caused by blue light (Ruebsam et al., 2018). To assess whether blue light exposure affects crystallin expression, we examined the levels of *crystallin* mRNAs in the retinal tissues of rats exposed to blue light. As a result, we observed a significant decrease in both *Cryaa* mRNA and protein levels (Fig. 2A and B). Furthermore, immunohistochemistry allowed for a more detailed analysis of CRYAA protein expression. As shown in Figure 2C and D, immunoreactivity for CRYAA significantly decreased in the retina exposed to blue light, including the ganglion cell layer and outer segments. Additionally, R-28 cells exposed to blue light exhibited a significant decrease in *Cryaa* mRNA levels (Fig. 2E). Moreover, blue light exposure precisely downregulated the expression of CRYAA protein in R-28 cells (Fig. 2F). These results suggest that the level of CRYAA is involved in the development of neuro-retinal cell degeneration caused by blue light.

### Regulation of CRYAA Expression by miR-325-3p

**Fig. 3:**
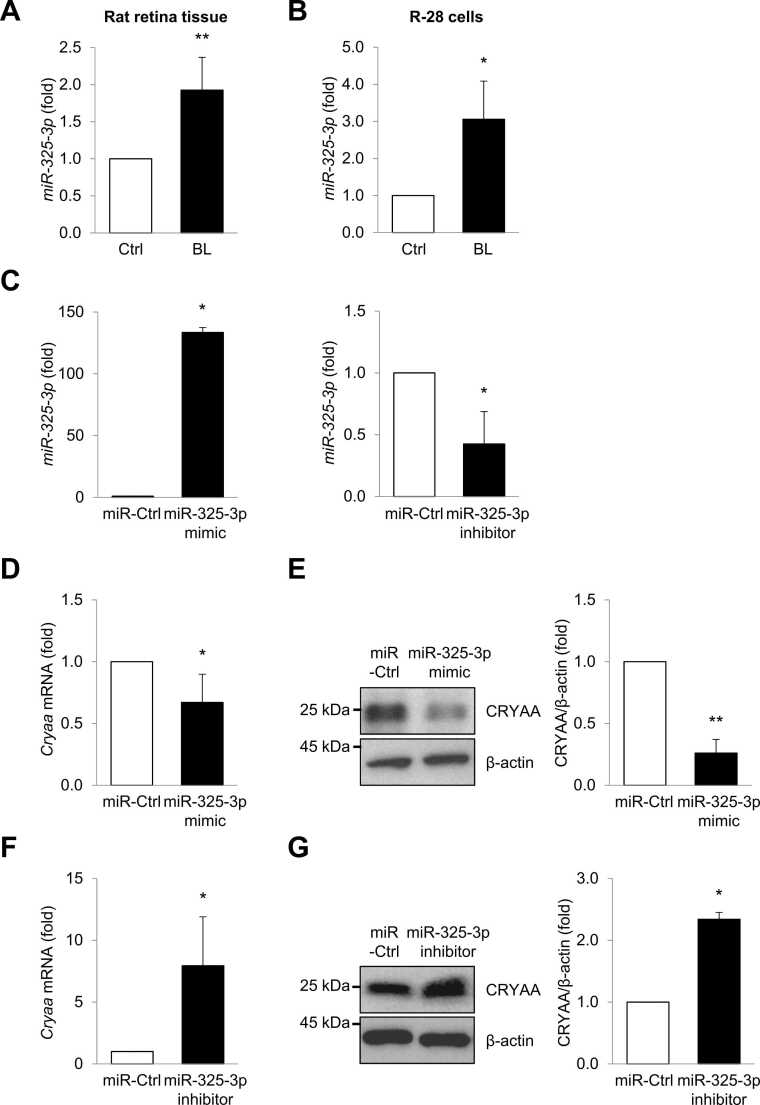
The miR-325-3p regulates *Cryaa* mRNA and protein expressions in neuro-retinal cells. (A, B) After blue-light exposure, miR-325-3p levels in the retinal tissues of rats and R-28 cells were quantified by RT-qPCR. (C) After transfection of R-28 cells with miR-325-3p mimic or inhibitor and appropriate control (miR-Ctrl), levels of miR-325-3p were quantified by RT-qPCR. Levels of *Cryaa* mRNAs (D, F) and CRYAA proteins (E, G) in R-28 cells were analyzed by RT-qPCR and western blotting, respectively. *U6* RNA, and *Gapdh* mRNA were used for normalization, and β-actin was used as a loading control. Data represent the means ± SEM from 3 independent experiments*.* *P < .05; **P < .01.

MiRNAs have the ability to specifically target mRNA, leading to post-transcriptional regulation. To identify the upstream regulator responsible for CRYAA downregulation, we employed a computational prediction algorithm (TargetScan) to identify potential miRNA that targets *Cryaa* mRNA. Interestingly, miR-325-3p was predicted as an exclusive regulator targeting the 3′UTR of *Cryaa* mRNA. To identify miRNA involved in the cause of retinal damage due to blue light, we quantified the levels of miR-325-3p in the retinal tissues of rats and R-28 cells exposed to blue light using RT-qPCR. Interestingly, the levels of miR-325-3p were significantly upregulated in both retinal tissues of rats and R-28 cells upon exposure to blue light (Fig. 3A and B). Thus, these results suggest that the expression of miR-325-3p might be associated with blue light-mediated gene regulation in neuro-retinal cells.

To elucidate whether the expressions of *Cryaa* mRNA and protein are regulated by miR-325-3p, we analyzed them by RT-qPCR and western blotting after transfection with miR-325-3p mimic or anti-miR-325-3p in R-28 cells (Fig. 3C-G). Ectopic expression of miR-325-3p mimic downregulated *Cryaa* mRNA levels and also decreased CRYAA protein expression in R-28 cells (Fig. 3D and E). Conversely, upregulation of *Cryaa* mRNA and protein levels was observed upon inhibition of miR-325-3p in R-28 cells and also increased CRYAA expression (Fig. 3F and G).

**Fig. 4:**
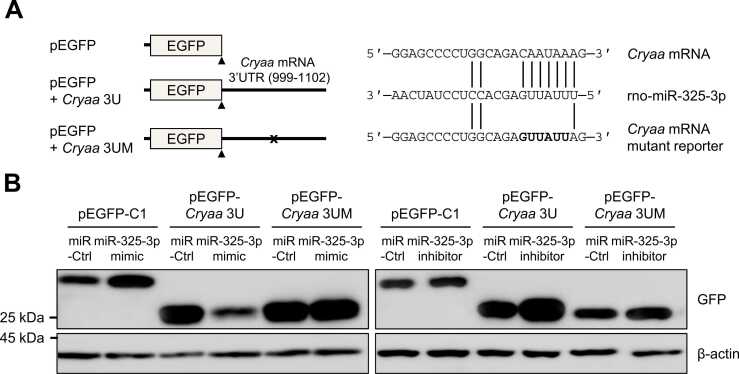
MiR-325-3p regulates *Cryaa* mRNA by targeting its 3′UTR. (A) Schematic of reporter constructs. 3′UTR of *Cryaa* mRNA containing a miR-325-3p binding site was inserted into pEGFP-C1. A mutant reporter construct lacking the miR-325-3p binding site was generated using site-directed mutagenesis. (B) After transfection to R-28 cells with miR-325-3p mimic, miR-325-3p inhibitor, and appropriate control (miR-Ctrl), together with each reporter plasmid, levels of GFP were assessed by western blotting. β-actin was used as a loading control. Data represent the means ± SEM from 3 independent experiments.

To further determine whether miR-325-3p could directly downregulate *Cryaa* mRNA, EGFP reporter constructs were generated by inserting the 3′UTR region of *Cryaa* mRNA (999-1102 bp) and by mutating miR-325-3p binding sites (Fig. 4A). As shown in Figure 4B, EGFP reporter expression (EGFP-*Cryaa*-3U) was downregulated by ectopic expression of miR-325-3p mimic, whereas the control EGFP reporter (EGFP-Cl) was not affected by miR-325-3p. On the contrary, inhibition of miR-325-3p upregulated EGFP reporter expression. Additionally, no significant changes were observed in mutant EGFP reporters by miR-325-3p mimic or inhibitor. Taken together, these results indicate that miR-325-3p contributes to the downregulation of *Cryaa* mRNA and protein levels in blue light-induced neuro-retinal degeneration.

### Prevention of CRYAA for Blue Light-induced Neuro-retinal Cell Death

**Fig. 5:**
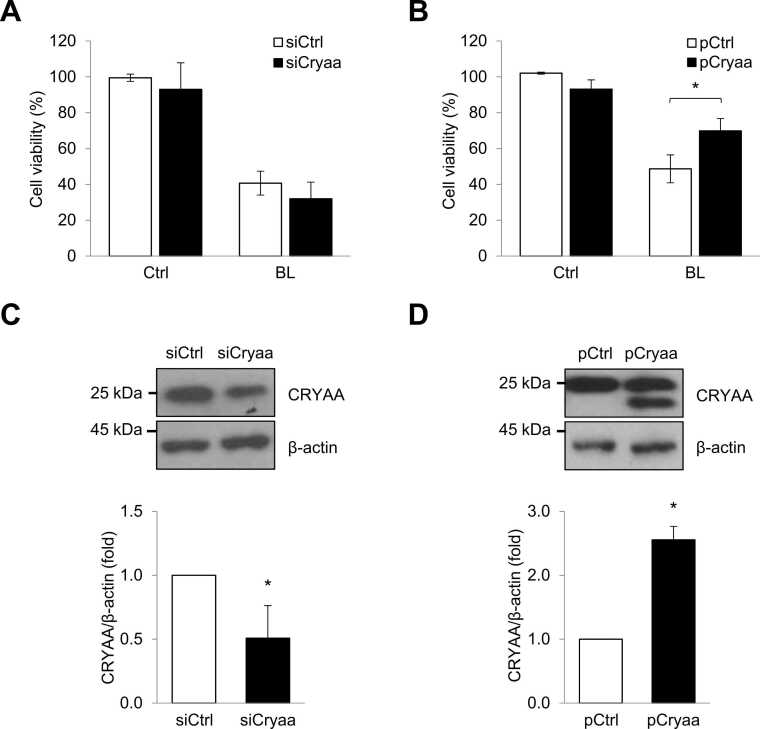
Blue light reduces neuro-retinal cell viability through *Cryaa* mRNA and miR-325-3p axis. (A) After transfection with siCryaa and appropriate control (siCtrl), the viability of R-28 cells exposed with or without blue light was determined by MTT assay. (B) After transfection with plasmid pCMV-Cryaa (pCryaa) and appropriate control (pCtrl), the viability of R-28 cells exposed with or without blue light was determined by MTT assay. (C, D) Representative levels of CRYAA protein in R-28 cells (transfected with siRNA or plasmid) were determined by western blotting. β-actin was used as a loading control. Data represent the means ± SEM from 3 independent experiments. *P < .05.

Blue light exposed neuro-retinal cells significantly deteriorated cell viability (Fig. 1). To assess the influence of CRYAA on neuro-retinal cell viability, we transfected siRNA to silence *Cryaa* or plasmid to overexpress CRYAA in R-28 cells (Fig. 5C and D) and then exposed the cells to blue light. The blue light-induced cell viability of R-28 cells was not affected by the knockdown of *Cryaa*, whereas CRYAA overexpression significantly alleviated cell viability (Fig. 5A and B). Taken together, these results suggest that the regulation of miR-325-3p/CRYAA axis plays a crucial role in the survival and function of neuro-retinal cells during the development of retinal degeneration caused by blue light.

## DISCUSSION

The retina is a critical component of the eye responsible for processing vital visual information, making it an area of significant interest in eye research. This study elucidated the impact of blue light exposure on important eye structures, particularly the retina. Our study revealed that blue light exposure leads to degenerative cell death in the retinal tissue of rats and cultured neuro-retinal cells. Furthermore, we observed a significant downregulation of CRYAA in response to blue light exposure, accompanied by an increase in miR-325-3p levels. We presented evidence that miR-325-3p promotes blue light-induced neuro-retinal cell death by targeting *Cryaa* mRNA. Moreover, we highlighted the pivotal role of CRYAA in ameliorating neuro-retinal cell death caused by blue light exposure.

Ever since the first report of retinal injury caused by blue light in 1978 (Ham et al., 1978), numerous researchers have investigated the photochemical damage to the retina. It has been suggested that exposure to blue light significantly increases the synthesis of reactive oxygen species, which in turn leads to the loss of photoreceptor cells, inflammation, and apoptotic cell death (Ouyang et al., 2020). Blue light-induced retinal degeneration has been extensively studied, both in vivo and in vitro. Rodents exposed to blue light have shown a remarkable reduction in the thickness of the outer nuclear layer, apoptosis of photoreceptors and retinal neurocytes, as well as microglia activation (Chen et al., 2019, Hu et al., 2016, Shang et al., 2017). Furthermore, various retinal cells, including human retinal pigment epithelium cells (ARPE-19), murine photoreceptor-derived cells (661W), mouse neuronal precursor cells (RGC-5), and rat retinal precursor cells (R-28), have also been observed to undergo apoptotic cell death as a result of blue light exposure (Huang et al., 2014, Li et al., 2018a, Sparrow and Cai, 2001, Zhang et al., 2012). In the present study, we expand on the understanding of blue light-induced retinal cell death by showing that miR-325-3p levels are significantly upregulated in the retinal tissues and R-28 cells following blue light exposure, which correlates with the downregulation of CRYAA.

Recent studies have expanded our understanding of crystallins, revealing their presence not only in the lens but also in various ocular and nonocular tissues (Srinivasan et al., 1992). Notably, α-crystallins, a major subgroup within the crystallin family, have been associated with chaperone functions and the inhibition of apoptosis (Wang et al., 2021). Consequently, α-crystallins play a crucial role in safeguarding RPE against injury induced by oxidative and endoplasmic reticulum stress, while also influencing autophagy processes (Kannan et al., 2016). In the present study, we investigated the role of CRYAA in neuro-retinal cells damaged by blue light. We revealed that the downregulation of *Cryaa* mRNA and protein levels is deeply involved in blue light-mediated neuro-retinal cell death. As depicted in Figure 5, exogenously expressed CRYAA exhibited the potential to protect these cells from blue light-induced cell death. CRYAA’s chaperone activity is particularly important in the retina, where cells are continuously exposed to oxidative stress and blue light-induced damage. CRYAA contributes to maintaining cellular homeostasis under diverse stress conditions by preventing the aggregation of misfolded proteins and protecting cells from stress-induced apoptosis (Andley, 2007). Additionally, CRYAA can inhibit apoptosis by interacting with apoptotic factors such as caspases (Fort and Lampi, 2011), stabilize the cytoskeleton and cellular membranes, and act as an antioxidant to reduce reactive oxygen species levels generated under blue light exposure. Our findings strongly suggest that crystallin proteins, particularly CRYAA, are indispensable for maintaining the viability and functionality of neuro-retinal cells under external stress conditions.

It has been studied that the expression of crystallins is tightly regulated at the transcriptional, post-transcriptional, and translational levels (Cvekl and Eliscovich, 2021). Understanding the regulatory mechanisms of CRYAA expression is necessary for developing strategies to modulate its expression or activity protecting against retinal degeneration induced by blue light exposure. In this study, we first identified a potential miRNA, miR-325-3p, capable of targeting *Cryaa* mRNA by employing computational prediction algorithms such as TargetScan.

MiRNAs, which are small single-stranded noncoding RNAs typically composed of 18 to 25 nucleotides, exert their regulatory functions by binding to complementary sites within the 3′UTR of target mRNAs. This interaction can lead to mRNA degradation or inhibition of mRNA translation. Numerous studies have revealed the critical roles of miRNAs in various biological and disease processes, encompassing metabolism, proliferation, differentiation, development, migration, and apoptosis (Lu and Liston, 2009). MiRNAs have also emerged as key players in ophthalmologic diseases. For example, miR-15b, which targets *VEGFa* mRNA, exhibits downregulation in the retinas of patients with proliferative diabetic retinopathy (Yang et al., 2020). Additionally, human RPE cells isolated from age-related macular degeneration donors have demonstrated decreased levels of miR-184, impacting RPE phagocytic function through enhanced expression of ezrin (Murad et al., 2014). Moreover, in an N-methyl-D-aspartate-induced glaucoma model, miR-93-5p has been shown to suppress autophagic cell death in retinal ganglion cells by inhibiting the expression of PTEN (Li et al., 2018b). These findings underscore the diverse and crucial roles of miRNAs in ophthalmologic diseases, making them attractive candidates for further research and therapeutic exploration.

The results of our study revealed an upregulation of miR-325-3p in neuro-retinal cells damaged by blue light, both in the retinal tissue of rats and in cultured cells (Fig. 3A and B). Additionally, we observed a significant inverse correlation between miR-325-3p and CRYAA expression (Fig. 3D-G). EGFP reporter analysis further demonstrated that *Cryaa* mRNA is a direct target of miR-325-3p (Fig. 4). While we also observed differential regulation of other *crystallin* mRNAs in blue light-exposed neuro-retinal cells, further studies are needed to investigate the regulatory mechanisms associated with miR-325-3p for these crystallins. Furthermore, it is necessary to explore the other miRNAs responsible for the regulation of crystallin expression under blue light exposure.

In summary, this study illustrates the detrimental impact of blue light on retinal health and the potential role of CRYAA and miR-325-3p in mediating this effect. Our findings suggest that CRYAA and miR-325-3p may serve as valuable biomarkers and potential treatment targets for retinal degeneration induced by blue light. Understanding these findings and their implications for retinal function will drive future research toward the development of effective treatments and preventive measures for retinal degeneration caused by blue light exposure.

## Author Contributions

**Young-Hoon Park:** Conceptualization, Funding acquisition, Project administration, Supervision, Writing – review & editing. **Chongtae Kim:** Conceptualization, Project administration, Validation, Writing – review & editing. **Subeen Oh:** Conceptualization, Investigation, Validation, Visualization, Writing – original draft.

## Declaration of Competing Interests

The authors declare that they have no known competing financial interests or personal relationships that could have appeared to influence the work reported in this paper.

## Data Availability

The data that support the findings of this study are available from the corresponding author upon reasonable request.
